# Damage Characteristics Analysis of Laser Ablation Triple-Junction Solar Cells Based on Electroluminescence Characteristics

**DOI:** 10.3390/s24154886

**Published:** 2024-07-27

**Authors:** Wei Guo, Jifei Ye, Hao Chang, Chenghao Yu

**Affiliations:** State Key Laboratory of Laser Propulsion & Application, Department of Aerospace and Technology, Space Engineering University, Beijing 101416, China; guoweikl@126.com (W.G.); changhao5976911@163.com (H.C.); yuchenghao0536@163.com (C.Y.)

**Keywords:** triple-junction solar cell, laser irradiation, work characteristics, electroluminescence characteristics

## Abstract

To study the physical property effects of the laser on GaInP/GaAs/Ge solar cells and their sub-cell layers, a pulsed laser with a wavelength of 532 nm was used to irradiate the solar cells under various energy conditions. The working performance of the cell was measured with a source meter. The electroluminescence (EL) characteristics were assessed using an ordinary and an infrared camera. Based on the detailed balance theory, in the voltage characteristics of an ideal pristine cell, the GaInP layer made the most significant voltage contribution, followed by the GaAs layer, with the Ge layer contributing the least. When a bias voltage was applied to the pristine cell, the top GaInP cell emitted red light at 670 nm, the middle GaAs cell emitted near-infrared light at 926 nm, and the bottom Ge cell emitted infrared light at 1852 nm. In the experiment, the 532 nm laser wavelength within the response spectrum bands of the GaInP layer and the laser passed through the glass cover slip and directly interacted with the GaInP layer. The experimental results indicated that the GaInP layer first exhibited different degrees of damage under laser irradiation, and the cell voltage was substantially attenuated. The GaInP/GaAs/Ge solar cell showed a decrease in electrical and light emission characteristics. As the laser energy increased, the cell’s damage intensified, gradually leading to a loss of photoelectric conversion capability, the near-complete disappearance of red light emission, and a gradual degradation of near-infrared emission properties. The EL imaging revealed varying damage states across the triple-junction gallium arsenide solar cell’s sub-cells.

## 1. Introduction

In recent years, the rapid development of the aerospace industry has brought widespread attention to solar cells, which are crucial energy storage components on spacecraft. Since solar cells serve as the primary power source for spacecraft, changes in their operating conditions can directly impact the spacecraft’s performance. However, the regular operation of solar cells in the working environment often encounters challenges from various factors, including single-particle effects, total dose effects, and displacement damage effects [1].

Among various factors, the Single Event Effect (SEE) is one of the primary causes of anomalies and failures in spacecraft induced by the space environment, primarily affecting semiconductor electronic devices such as solar cells. To simulate the single particle effects in devices within a space environment, laboratories commonly use lasers to conduct radiation experiments on semiconductor materials, studying the impact of material damage on the operational characteristics of the devices. Ma et al. utilized nanosecond, picosecond, and femtosecond pulsed laser irradiation experiments as analysis methods for the SEE of semiconductor materials and for providing a quantitative evaluation of sensitivity to these effects [2]. Their pulse laser experiments offered data support for using Si-based semiconductor devices in space exploration payloads. Ngom et al. employed femtosecond pulsed lasers to irradiate GaN-based semiconductor materials, discussing the relationship between laser wavelength and material penetration depth [3]. Zhu et al. used continuous lasers to irradiate GaAs-based semiconductor materials, analyzing the surface morphology of the irradiated materials through optical microscopy and the composition of the laser-induced products via X-ray photoelectron spectroscopy (XPS) [4]. Li et al. conducted modeling and simulation to analyze the electrical characteristics of multi-junction GaAs-based semiconductor materials under laser irradiation, finding that differences in laser energy directly influenced the material’s open-circuit voltage and, ultimately, its energy conversion efficiency [5].

When irradiating solar cells, a laser—a high-brightness light source—can easily disturb the cells [6,7]. If the laser energy density is sufficiently high, it can disrupt the cells’ ability to maintain regular operation, impacting the spacecraft’s performance. Therefore, researching solar cells’ damage process and characteristics under laser irradiation holds significant importance. Numerous scholars have conducted extensive research to investigate the impact of laser irradiation on cell performance and analyze the characteristics of cell damage. Recent studies on the laser irradiation of solar cells, including Si, GaAs, and GaInP/GaAs/Ge, encompassed experiments conducted in vacuum or atmospheric environments using pulsed or continuous wave (CW) lasers [4,7,8,9,10,11,12]. These studies aimed to investigate the effects of laser parameters on cell performance. Various instruments, such as optical microscopes, thermal imaging cameras [11], scanning electron microscopes (SEM) [12], laser-beam-induced current imaging (LBIC) [13], and X-ray photoelectron spectroscopy (XPS) [14], were employed to analyze mechanical, electrical, optical, thermal, and acoustic properties. The analyses included surface morphology measurements, electrical characteristics, temperature, and electroluminescence (EL) [15]. The damage characterization of solar cells is challenging to assess with ordinary methods, especially for multi-junction cell power source systems. Therefore, existing studies still need to thoroughly analyze the damage characteristics of laser-irradiated solar cells and their sub-cells. Given the close correlation between solar cell performance and damage characteristics, a comprehensive investigation into the impact of laser-induced damage on the solar cells and their sub-cells is of utmost importance.

EL diagnostics is a widespread technique for visually detecting defects within solar cell analysis systems [15]. The method utilizes an external power supply and cameras that can detect light emitted from the cells to spatially map out the local voltage variation of cells to capture the cell images. This study employed a 532 nm pulsed laser to irradiate GaInP/GaAs/Ge triple-junction solar cells, allowing for an analysis of EL properties of different sub-cells during the irradiation process. Furthermore, the electrical characteristics of the cells were meticulously measured, and this study investigated the surface morphology changes before and after laser irradiation. This research also evaluated the impact of cell damage on the output performance, providing referable insights into the overall functionality of the solar cells. By exploring the alterations in surface morphology, electrical characteristics, and EL properties, this study contributes to a comprehensive understanding of the effects of laser-induced damage on solar cell performance. The findings will advance our knowledge of solar cell behavior under laser irradiation and provide valuable guidance for designing and optimizing future spacecraft solar cell systems.

## 2. Experimental System and Principle

**Figure 1:**
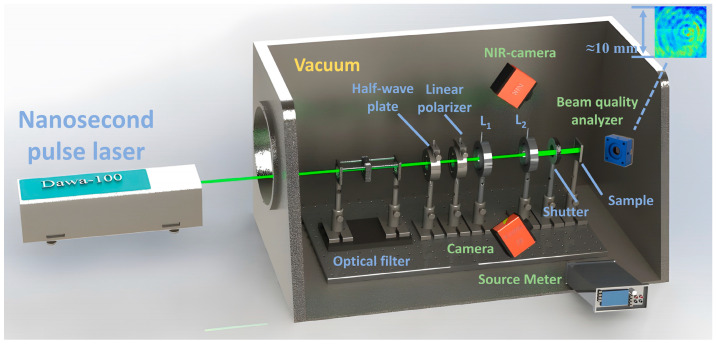
Laser irradiation experiment system.

The experiment used a laser irradiation source with a central wavelength of 532 nm to irradiate GaInP/GaAs/Ge solar cells. The anti-reflection coating (TiO_2_/Al_2_O_3_) on the solar cell’s surface has little effect on the 532 nm laser, and the laser can directly damage the solar cell. The experimental system, including a pulse laser, a polarization filter device, a beam expander, and a source meter, is shown in Figure 1. The polarization filter device consists of a half-wave plate and a linear polarizer. The beam expander device consists of two convex lenses, L_l_ and L_2_; the diameter of lens L_l_ is 25 mm, and its focal length is 50 mm, while the diameter of lens L_2_ is 25 mm, and its focal length is 100 mm. The spot size on the surface of the solar cell can be adjusted by changing the distance between lenses L_1_ and L_2_. The laser beam profiler (CinCam CMOS-1204, Cinogy, Göttingen, Germany) was used to measure the laser beam. And the area was about 0.785 cm^2^.

After laser irradiation, the optical microscope was used to analyze the surface morphology of the cell. Ordinary and near-infrared cameras were used to take EL images. Based on the surface topography, the source meter (Model 2450, Tektronix, Wilmington, DE, USA) was used to measure the working performance parameters of the cell. Finally, the influence of cell damage on the output performance under pulse laser irradiation was analyzed.

**Figure 2:**
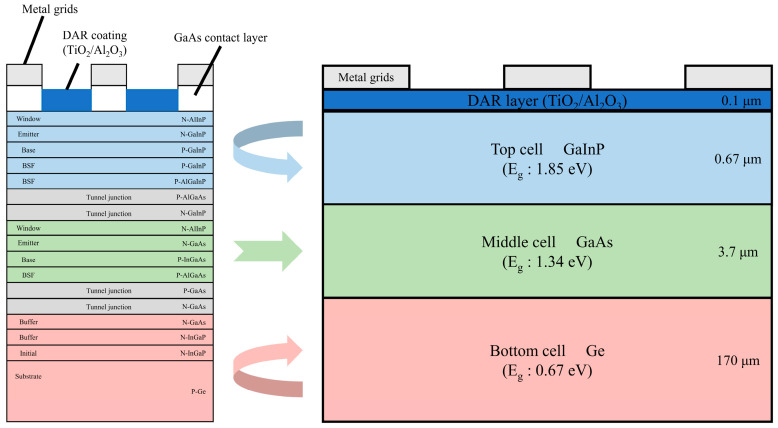
Structural model of GaInP/GaAs/Ge cell.

The physical structure of the GaInP/GaAs/Ge solar cells used in the experiments and their bandgap widths are shown in Figure 2. The cell is 10 mm × 10 mm in size and 175 μm thick. The cell structure mainly includes a double-layer anti-reflection coating (DAR), a top cell, a middle cell, and bottom cell layers. 

The response spectra of the sub-cell are 350~700, 700~880, and 880~1750 nm, respectively. The intrinsic properties of semiconductor materials fundamentally determine solar cells’ electroluminescence (EL) characteristics. Electrons within the semiconductor absorbed externally injected energy and became excited. Electrons release energy through light radiation when transitioning from an excited high-energy state to a lower-energy state [16,17]. Consequently, when a bias voltage was applied to the solar cell, the radiative recombination of non-equilibrium carriers in the material resulted in the emission of specific optical signals.

The photon energy emitted from the interband transitions in the sub-cell was directly related to the material’s bandgap energy (*E_g_*) [16,18]:

(1) $$E_{g}=\hbar \omega =\frac{hc}{\lambda }$$

where *E_g_* is the material’s bandgap energy, *h* is the Planck constant, *c* is the speed of light, A and λ is the wavelength of the photon.

**Figure 3:**
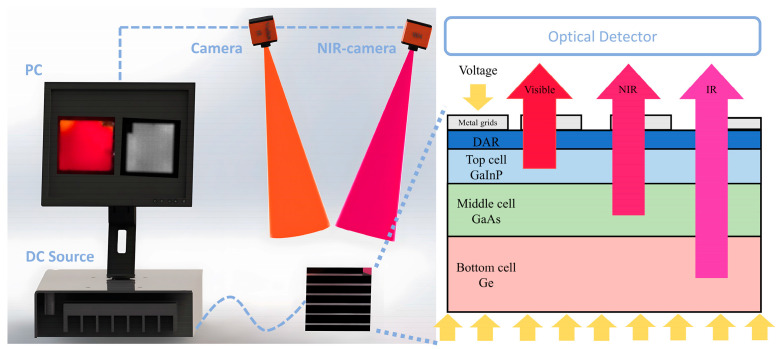
EL measurement experimental device.

A single-junction sub-cell was equivalently modeled as a single P-N junction light-emitting diode (LED), while a multi-junction cell was seen as multiple LEDs. Therefore, a GaInP/GaAs/Ge solar cell was a series connection of three sub-cells. The experimental setup for EL measurement is illustrated in Figure 3. Each sub-cell emitted photons of specific wavelengths when an external energy source was injected into the solar cell. The wavelengths are shown in Table 1.

In Table 1, under the condition of an applied bias voltage, the top GaInP sub-cell emitted red light with a wavelength of 670 nm, the middle GaAs sub-cell emitted near-infrared light with a wavelength of 926 nm, and the bottom Ge sub-cell emitted infrared light with a wavelength of 1852 nm.

In the experiment, the characteristic parameters of each sub-cell in the pristine solar cells were analyzed based on the detailed balance theory, a physical model that described the work of photovoltaic cells under ideal conditions. During measurements, the *E_g_* characterized the performance of photovoltaic materials.

Among the cell characteristics, the $V_{oc}$ parameter was of particular interest, as follows [19]:

(2) $$V_{oc}=\frac{kT}{q}\ln\left(\frac{qP_{\lambda }h^{3}c^{2}}{2\pi qkT{E_{g}}^{3}}\right)+\frac{E_{g}}{q}$$

where *q* is the electron charge; *k* is Boltzmann’s constant; *h* is Planck’s constant; *c* is the speed of light; *T* is the temperature of the cell material in the experimental measurement environment, which is approximately 300 K; *E_g_* is the bandgap energy of the material at room temperature; and $P_{\lambda }$ is the power density of the measurement light source.

**Figure 4:**
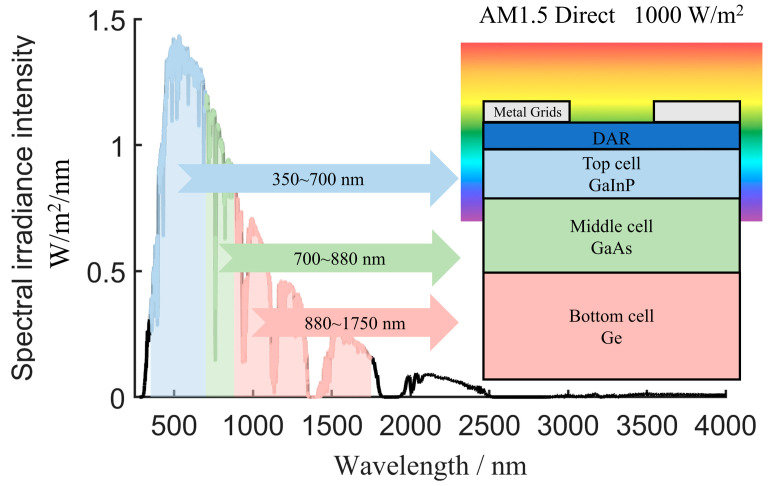
AM1.5 Direct electrical characteristic test environment under 1000 W/m^2^ light conditions.

As shown in Figure 4, the power density of the light source for each sub-cell layer is expressed as

(3) $$P_{\lambda_{1,2,3}}=\frac{\int_{\lambda_{1,2,3}}S\left(\lambda \right)d\lambda }{\int_{\lambda_{0}}S\left(\lambda \right)d\lambda }P_{0}$$

where $P_{0}$ is the irradiance intensity of the solar light source (AM1.5 D); $S\left(\lambda \right)$ is the spectral distribution function of the light source intensity; $\lambda_{0}$, $\lambda_{1}$, $\lambda_{2}$, and $\lambda_{3}$, respectively, represent the emission spectrum segment of the simulated light source and the spectral response segments of different sub-cells.

Figure 4 depicts the measurement environment for electrical characteristics. The analysis of the electrical properties of each sub-cell layer in the pristine solar cell is shown in Figure 4 and Table 1. The voltage contribution ratios of the ideal original GaInP, GaAs, and Ge sub-cells were approximately 3.46:2.34:1. This distribution aligned with the experimental measurements reported in [20], whereby the GaInP layer contributed the most voltage, followed by the GaAs layer, with the Ge layer contributing the least.

In the experiment, the optical signals emitted by the solar cell were captured using a camera. The camera-generated optical images were then transmitted to a computer. The damage characteristics of the triple-junction GaAs solar cell under laser irradiation were analyzed based on the EL properties of the cell.

## 3. Results

The experiment investigated the damage characteristics of triple-junction GaInP/Ga-As/Ge solar cells under pulsed laser irradiation. The solar cells used in the experiment had dimensions of 10 × 10 mm, with an irradiation spot diameter of approximately 10 mm. The irradiation experiments were conducted on the triple-junction GaAs solar cells. The cells were irradiated by the laser using different energy densities. The cells’ surface morphology, I–V characteristics, open-circuit voltage, and EL properties were measured and analyzed.

### 3.1. Effect of Laser Irradiation on Surface Damage Morphology and Electrical Characteristics of Cells

**Figure 5:**
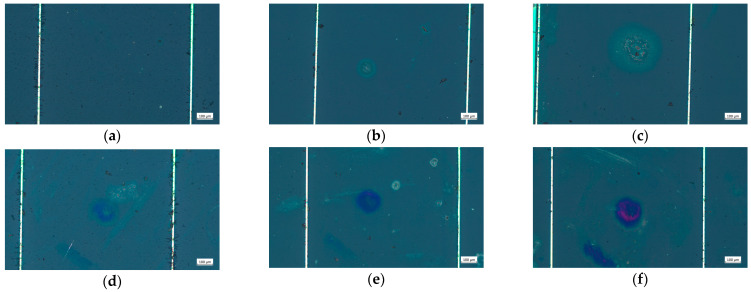
Microcosmic changes in the cell surface morphology irradiated by laser. (**a**) Unirradiated; (**b**) 0.031 J/cm^2^; (**c**) 0.058 J/cm^2^; (**d**) 1.166 J/cm^2^; (**e**) 1.75 J/cm^2^; (**f**) 5.832 J/cm^2^.

When the laser energy density reached 0.031 J/cm^2^, the surface morphology of the cell was revealed, as shown in Figure 5b. Noticeable circular ablation pits appeared on the surface of the cell, and the material of the cell underwent erosion. When the laser irradiation energy density reached 0.058 J/cm^2^, significant damage was observed on the cell surface, with the ablation-damaged areas having expanded.

When the laser energy density reached 1.166 J/cm^2^, a distinct ablation pit was observed at the center of the irradiated area on the cell surface, with colored ablation regions around the pit. With increasing laser power density, the damaging effect became more apparent. Upon 1.75 and 5.832 J/cm^2^, the colored ablation regions near the ablation pit were more pronounced.

The 532 nm laser wavelength was seen within the response spectrum bands of the GaInP layer. During laser irradiation, the laser energy underwent photoelectric conversion in the GaInP layer, with a portion of the unconverted energy being transformed into thermal energy. The damage to solar cells by nanosecond laser irradiation is primarily thermal. Rapid energy deposition leads to the melting and vaporization of cell materials at the center of the laser spot. The nanosecond duration of the laser pulse significantly exceeds the material’s energy relaxation time, so thermal diffusion is pronounced. After the laser pulse irradiation ceases, the ablated products quickly transition from molten to solid. The melting and re-solidification of ablated products and oxidation–reduction reactions occurring in surrounding areas due to thermal diffusion collectively form a peripheral annular region. These peripheral and central regions constitute the ablation pit formed by ablation. The higher the laser energy density, the larger the ablation pit and surrounding annular region.

**Figure 6:**
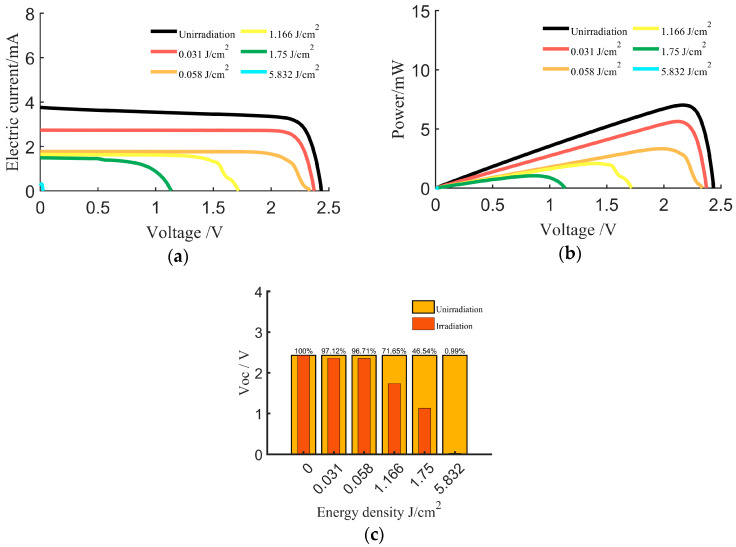
Variation curves of electrical characteristics of a triple-junction GaInP/GaAs/Ge cell irradiated by laser. (**a**) Voltage–current characteristics. (**b**) Power–voltage characteristics. (**c**) Open-circuit voltage V_oc_.

During the experiment, the electrical characteristics of the solar cell were measured using a Source Meter (2450), and the variations in the cell’s characteristic curves were analyzed. The effects of pulsed laser irradiation on the electrical characteristics are presented in Figure 6.

In Figure 6a, the abscissa is the voltage, in V, and the ordinate is the current, in mA; in Figure 6b, the ordinate is the power, in mW. In Figure 6c, the abscissa is the energy density, in J/cm^2^, and the ordinate is the voltage, in V. To study the influence of laser energy density on electrical performance parameters, the parameters were normalized relative to the original cell. The results are presented in Table 2.

In the experiment, when the laser energy density was 0.031 J/cm², it was observed that irradiation damage on the cell surface led to a degradation in performance (Figure 5b). The V_oc_ and I_sc_ exhibited a certain degree of decline, and the P_max_ slightly decreased. However, the shape of the curve remained broadly consistent with that of the original intact cell.

When the laser energy density was increased to 0.058 J/cm², it was observed that the damaged area on the cell surface expanded (Figure 5c). At this energy density, the V_oc_ decreased by 3.2%, and the P_max_ decreased by 52.6%. When the laser energy density was further increased to 1.166 J/cm², as shown in Figure 5d, ablation pits appeared in the central irradiated region of the cell surface, and material erosion was observed near the cell grid lines. Under these conditions, the V_oc_ decreased by 28.3%, and the P_max_ decreased by 52.6%

With further increases in laser energy density to 1.75 and 5.832 J/cm², the extent of cell damage intensified. At this stage, the laser caused irreversible damage to the cell’s work performance. The open-circuit voltage, maximum output power, and short-circuit current characteristics disappeared, and the cell’s photoelectric conversion ability was lost.

### 3.2. Effect of Laser Irradiation on EL Characteristics

When analyzing the damage characteristics of the solar cells under pulsed laser irradiation, the damage to each sub-cell layer significantly affected the cell’s overall performance.

**Figure 7:**
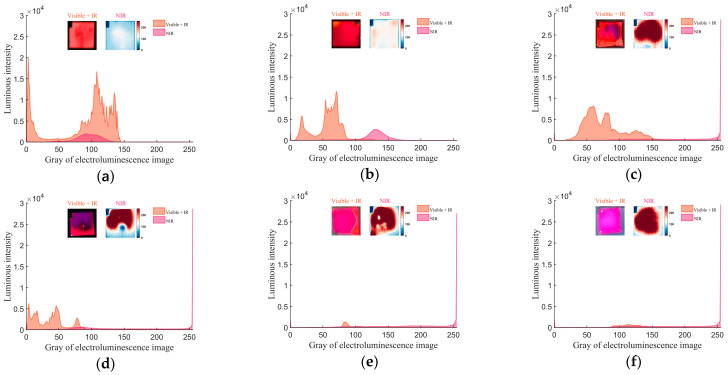
Electroluminescence changes in the triple-junction GaInP/GaAs/Ge cell irradiated by nanosecond laser. (**a**) Electroluminescence results with no laser ablation. (**b**–**f**) Electroluminescence results by laser irradiation when laser energy density is 0.031, 0.058, 1.166, 1.75, 5.832 J/cm^2^, respectively.

In the experiment, a bias voltage was applied to the cell using a DC power supply. EL images of the cell were captured using an ordinary camera and a near-infrared camera equipped with a 900~1100 nm notch filter. The ordinary camera captured optical information from both visible and near-infrared light emitted by the cell, while the near-infrared camera captured information precisely from the near-infrared light. The changes in the cell’s EL characteristics during laser irradiation are presented in Figure 7.

The figure utilizes the grayscale values from the electroluminescence of solar cells to represent their luminescence intensity under various degrees of damage, thereby assessing the damage characteristics of the solar cells. In Figure 7, the abscissa is the image gray value, and the ordinate is the frequency of occurrence of each grayscale value in the image recorded. The small image on the left displays the EL image captured by the ordinary camera in the visible and near-infrared spectra. The small image on the right shows the pseudocolor EL image in the near-infrared spectrum. The orange curve represents the grayscale distribution information of images taken by an ordinary camera, corresponding to the image on the left; the pink curve represents the grayscale distribution information of images taken by an infrared camera, corresponding to the pseudocolor image on the right. When the solar cell was undamaged, the GaInP layer cell exhibited the highest red luminescence intensity. After laser irradiation, the red light luminescence intensity decreased, and the grayscale distribution shifted to the left. The underlying GaAs layer corresponded to increased near-infrared luminescence intensity, with a rightward shift in grayscale distribution. Analysis of EL results revealed that the luminescence intensity of the cell decreased with the rise in laser energy density. The EL images could reveal the damaged area of the cell. When the laser energy density was low, the damaged area was primarily distributed around the laser-irradiated area. As the laser energy density increased, the impact area gradually expanded, eventually spreading to the entire cell. The electroluminescent image results suggested that even though the laser only ablated a part area on the cell surface, the damage could affect a large area of the solar cell.

As shown in Figure 7a, the characteristics of the EL of the pristine cell were measured. The image, captured by an ordinary camera, displayed a bright red visible light emission zone, while the near-infrared camera image showed a partial near-infrared light emission zone. As shown in Figure 7b, when the cell was irradiated with a laser energy density of 0.031 J/cm^2^, the intensity of the red visible light decreased with a leftward shift in the grayscale value curve, and the near-infrared emission intensity increased with a rightward shift in the grayscale value curve. According to Figure 5c, the erosion of the GaInP material, which emits visible red light at the laser irradiation center, caused a slight attenuation in the luminescence characteristics of the GaInP material in the irradiated area.

As shown in Figure 7e, at a laser energy density of 0.058 J/cm^2^, the center of the red visible light region exhibited a void, with a distinct near-infrared luminescence detected in that area. Figure 5c indicates significant damage on the surface of the GaInP material in the laser-irradiated central region. Following the damage and loss of the GaInP material, the camera could directly detect near-infrared light emissions from the contiguous GaAs material. In Figure 7e, near-infrared luminescent characteristics in the damaged GaInP area were significantly more potent than in undamaged areas. There was a noticeable decrease in the V_oc_ and the P_max_. The GaInP layer, which has a substantial voltage contribution, had lost part of its functionality.

As the laser energy density increased to 1.166, 1.75, and 5.832 J/cm^2^, the red luminescent region in the camera images gradually disappeared, yet near-infrared luminescence persisted at the locations where the red light was previously observed. The grayscale curve for red light shifted leftward and eventually vanished, while the grayscale curve for near-infrared light remained and shifted rightward. The area of damage on the cell surface expanded. Under the laser’s influence, internal cell materials sustained damage, particularly the GaInP material, which exhibited a worsening degradation in its ability to emit red visible light, eventually losing this capability. When the GaInP material lost its red luminescence characteristics, the GaInP layer had almost lost its working functionality. The adjacent GaAs material still retained some near-infrared luminescence properties. The cell had lost their photovoltaic conversion capability.

## 4. Conclusions

This experiment investigated the impact of GaInP/GaAs/Ge solar cells caused by the irradiation from a 532 nm pulsed laser on their operational performance. The electrical characteristic parameters of the cells were measured using a 2450 source meter. EL images were captured using an ordinary camera and an infrared camera to ascertain the extent of damage in different sub-cell layers based on their EL characteristics.

In the experiment, the laser wavelength was set at 532 nm, within the response spectrum range of the GaInP top cell layer. The laser irradiation passed through the anti-reflection layer and directly irradiation the GaInP layer. At a laser energy density of 0.031 J/cm^2^, the surface material of the cell began to erode, leading to performance degradation compared to the pristine cell. The GaInP layer sustained mild damage, decreasing the intensity of the red light emission. With the laser energy density increased to 0.058 J/cm^2^, the damaged area on the cell surface expanded, and the V_oc_ dropped. The damage to the GaInP layer became more severe, with the center of the red visible light region missing and a noticeable near-infrared luminescence detectable in that area. As the laser energy density further escalated, the damage to the cell increased, becoming more pronounced, with the cell’s electrical properties gradually fading. At laser energy densities of 1.166, 1.75, and 5.832 J/cm^2^, the GaInP layer was almost completely damaged, with the GaAs layer exhibiting less severe damage. The red luminescent region progressively vanished, yet near-infrared luminescence was still present at the locations where the red light had disappeared.

In summary, the study analyzed the relationship between the EL characteristics of each sub-cell layer in a solar cell and its damage characteristics under laser irradiation. The findings confirmed the cell’s damage characteristics and EL properties varied under different laser energy densities. This research has explored the impact of factors such as laser wavelength on cell characteristics, offering insights into the threshold for laser irradiation damage in future studies and providing ideas for cell protection and structural optimization.

## Figures and Tables

**Table 1:** GaInP/GaAs/Ge solar cell material properties.

Sub-Cell Material	Response Spectral Band (nm)	Band Gap *E_g_* (eV)	The Material Emits Photon Wavelength (nm)
GaInP	350~700	1.85	670
GaAs	700~880	1.34	926
Ge	880~1750	0.67	1852

**Table 2:** Effect of laser irradiation on output performance of cell.

Energy Density (J/cm^2^)	Unirradiation	0.031	0.058	1.166	1.75	5.832
V_oc_	1	97.13%	96.71%	71.65%	46.54%	0.99%
I_sc_	1	71.84%	47.97%	43.16%	39.18%	8.26%
P_max_	1	80.10%	47.39%	29.60%	14.84%	0.06%
Fill Factor (FF)	1	87.20%	77.58%	72.71%	61.82%	54.41%

## Data Availability

Data are contained within the article.
